# Cardiotoxicity of Chemical Substances: An Emerging Hazard Class

**DOI:** 10.3390/jcdd9070226

**Published:** 2022-07-14

**Authors:** Nikolaos Georgiadis, Konstantinos Tsarouhas, Jean-Lou C. M. Dorne, George E. N. Kass, Petroula Laspa, Konstantinos Toutouzas, Elisabeth A. Koulaouzidou, Dimitrios Kouretas, Christina Tsitsimpikou

**Affiliations:** 1 European Chemicals Agency, 00150 Helsinki, Finland; nikolaos.georgiadis@echa.europa.eu; 2 Department of Biochemistry & Biotechnology, University of Thessaly, 38221 Larissa, Greece; petroulalas17@gmail.com (P.L.); chtsitsi@yahoo.com (C.T.); 3 Department of Cardiology, University Hospital of Larissa, 41110 Larissa, Greece; ktsarouhas14@yahoo.gr; 4 European Food Safety Authority, 43126 Parma, Italy; jean-lou.dorne@efsa.europa.eu (J.-L.C.M.D.); georges.kass@efsa.europa.eu (G.E.N.K.); 5 First Department of Cardiology, Hippokration Hospital, Medical School, University of Athens, 11527 Athens, Greece; ktoutouz@gmail.com; 6 Division of Dental Tissues’ Pathology and Therapeutics (Basic Dental Sciences, Endodontology and Operative Dentistry), School of Dentistry, Aristotle University Thessaloniki, 54124 Thessaloniki, Greece; koulaouz@dent.auth.gr; 7 Directorate of Energy, Industrial & Chemical Products, General Chemical State Laboratory of Greece, 11521 Athens, Greece

**Keywords:** anthracyclines, echocardiography, ejection fraction, fractional shortening, biochemical indices, creatine kinase-myocardial band isoenzyme, glutathione peroxidase, rats

## Abstract

(1) Background: Human health risks and hazards from chemical substances are well regulated internationally. However, cardiotoxicity, is not defined as a stand-alone hazard and therefore there are no defined criteria for the classification of substances as cardiotoxic. Identifying and regulating substances that cause cardiovascular adverse effects would undoubtedly strengthen the national health systems. (2) Methods: To overcome the aforementioned gap, a roadmap is proposed for identifying regulatory criteria from animal studies and endorse legislation in order to classify substances as cardiotoxic. The roadmap consists of: (i) the identification of the appropriate animal species and strains; (ii) the identification of the lines of scientific evidence (e.g., histopathological, biochemical and echocardiographic indices etc.) from animal studies with relevance to humans; (iii) the statistical analysis and meta-analysis for each line of scientific evidence after exposure to well-established cardiotoxicants to humans (e.g., anthracyclines) in order to identify threshold values or range of normal and/ or altered values due to exposure; (iv) validation of the above described lines of evidence in animals exposed to other alleged cardiotoxic substances (e.g., anabolic androgen steroids (AAS) and pesticides); (v) establishment of mechanisms of action based on information of either known or alleged cardiotoxicants; and (vi) introduction of novel indices and in silico methods. (3) Results: Preliminary results in rats indicate a clear distinction from normal values to values measured in rats exposed to anthracyclines regarding left ventricle (LV) fractional shortening (FS) and LV ejection fraction (EF). A distinctive pattern is similarly observed for Creatine Kinase-Myocardial Band isoenzyme (CK-MB) and cardiac tissue glutathione (GSH). These findings are encouraging and indicate that there is room for targeted research to this end, and that these specific indices and biochemical markers should be further investigated in order to be developed to regulatory criteria. (4) Conclusions: Further research should be conducted by both the scientific and regulatory community that aims to clearly define the cardiotoxicity hazard caused by chemicals and develop a full set of scientific criteria.

## 1. Introduction

Human health risks and hazards from chemical substances are well regulated internationally. When the available data meet the established classification criteria for the different regulated hazards as they are defined by the international legislations, a certain hazard class and category are assigned accordingly. Classification criteria generally include well defined endpoints, reference/threshold values from animal studies with relevance to humans, along with criteria for epidemiological and clinical data from various populations. Special provision with regards the criteria development for classification is given to vulnerable population groups, such as workers, pregnant women and children etc. The hazard classes in general cover physical, environmental and human health hazards. It should be noted that all chemical substances, pharmaceutical active substances and biocides included, fall within the scope of harmonized classification. More specifically for human health hazards, the most important classification hazard classes are listed below:

○ Acute toxicity (oral, dermal, inhalation);
○ Skin corrosion/skin irritation;
○ Serious eye damage/eye irritation;
○ Respiratory sensitization;
○ Skin sensitization;
○ Mutagenicity;
○ Carcinogenicity;
○ Toxicity for reproduction;
○ Specific target organ toxicity (STOT) (single exposure, SE);
○ Specific target organ toxicity (STOT) (repeated exposure, RE);
○ Aspiration hazard.

However, cardiotoxicity is not defined as a stand-alone hazard and therefore there are no defined criteria for the classification of substances as cardiotoxic. Hence, from a regulatory point of view, the identification and regulation of substances that cause cardiovascular adverse effects cannot be enforced and among others an opportunity to strengthen the national health systems remains unused. It has been estimated that at least 23% of all cardiovascular pathologies could be attributed to environmental exposures, mainly chemicals. Nevertheless, the causative agents remain largely uncharacterized [1].

More specifically, in the Classification Labelling and Packaging (CLP) European Regulation (Reg 1272/2008/EC), cardiotoxicity may be partially covered within the STOT hazard class based only on expert judgment of the evaluator toxicologist, since specific criteria are not available in order to assess findings from animal studies or early clinical manifestations in humans. For other human organs, STOT criteria have been developed within the framework of CLP for toxic damage caused by chemicals, such as lungs, liver, kidneys, endocrine system, etc. It must be noted that no animal testing method for cardiotoxicity is currently available in the Organization for Economic Co-operation and Development (OECD) Testing Guidelines and cardiovascular measurements are not included in the current evaluation programs of environmental chemicals [1]. Hence, potentially cardiotoxic substances or products are not restricted at a regulatory level.

Specific classification criteria should be used in a weight-of-evidence approach for the assessment of cardiotoxicity of chemicals and, thus, reduce cardiovascular adverse effects in the general population after exposure to chemicals.

Classification should be based on the following scientific evidence (findings), in a way that would reduce uncertainties:

- Anatomical and histopathological data;
- Echocardiographic data on contractility (e.g., LVEF, LVFS), documentation of cardiac frequency and/or implementation of other cardiac imaging modalities (e.g., MRI);
- Biochemical data, of generic nature (e.g., circulating oxidative stress markers), of more specific nature (e.g., oxidative stress markers of the cardiac tissue) and heart specific biomarkers (e.g., cardiac enzymes);
- Identification of pathways and mode of actions, which modulate the changes observed in different parameters after exposure to chemicals;
- In silico data, such as adverse outcome pathways (AOPs), omics, in vitro, organs on a chip, physiologically based pharmacokinetic models (PBPK), etc.

The authors, after having recognized the regulatory gap in describing cardiotoxicity as a separate hazard class for chemicals, try to describe a methodological approach (suggested roadmap) to the collection of animal evidence to be applied in the future by regulators and scientists in order to identify cardiotoxic chemicals from a regulatory perspective and, consequently, endorse legislation measures to protect human health from relevant exposure. In this context, the authors discuss respective preliminary results obtained from their research group (published and unpublished data) on specific indices and biochemical markers showing that they should be further investigated in order to set regulatory criteria and highlight the need for targeted research to this end.

## 2. Current Definition of Cardiotoxicity

Cardiotoxicity has so far been mainly linked to side effects in humans after the use of pharmaceuticals and it can be diagnosed in individuals post-exposure, at the time of established clinical manifestations, at which stage could even be irreversible [2,3,4,5,6]. The most common class of drugs known for cardiotoxic side-effects is anthracyclines.

Anthracyclines are used in cancer therapy and they are isolated from *Streptomyces* bacterium. They are used for treating different cancers, including leukemias, lymphomas, as well as breast, stomach, uterine, ovarian, bladder cancer, and lung cancers [7,8,9]. The first anthracycline discovered was daunorubicin. It is produced by an actinobacterium, *Streptomyces peucetius*. Clinically, the most important anthracyclines are doxorubicin, daunorubicin, epirubicin and idarubicin. Anthracyclines are well-known to cause myocardial suppression in patients going through the aforementioned therapies. In this sense, they are used in animal studies as an applicable method to introduce a model of dilated cardiomyopathy [10].

Cardiotoxicity is not restricted to anticancer agents. Chronically administered drugs, such as neurologic/psychiatric agents, also represent a major problem because cardiotoxicity may become evident only after long-term accumulation of the drug or its metabolites. Strikingly, almost 10% of drugs in the last four decades have been recalled from the clinical market worldwide due to cardiovascular safety concerns [11]. Currently, assessing the cardiotoxicity potential is a crucial parameter in drug development.

In humans, cardiotoxicity describes the deterioration of myocardial function post cancer treatment manifested by myocardial dysfunction and, in several cases, overt symptoms of heart failure. Echocardiography is the standard method to ascertain the presence of cardiotoxicity which can be manifested as acute, early or late. The cut-off values of echocardiographic indices in humans for the identification of cardiotoxicity caused by chemotherapeutics differ between the American and European guidelines: the definition considers a lower cut-off value of normality for the left ventricle ejection fraction (LVEF) of 50% in Europe [12] and 53% in the USA [13]. It should be stressed that these scientific guidelines indicate that a drop of LVEF compared to the patient’s previous values is also required. This is of high importance since patients presenting this decline in cardio-imaging indices of cardiac function should be treated with angiotensin converting enzyme inhibitors (ACEIs) or angiotensin II receptor blockers (ARBs) in combination with beta-blockers [14]. However, there is a debate among the experts related to possible modifications of anticancer treatment in patients who meet the aforementioned criteria. The above-described definition of cardiotoxicity post-cancer treatment, for simplicity reasons, does not account for indices of myocardial injury or specific cases of cardiovascular disease of different origin, such as coronary artery disease, despite the growing evidence for an etiological association with cancer treatment [12]. Nevertheless, assessing drug-induced cardiotoxicity risk including QT interval prolongation, for example, is nowadays considered an integral part of the standard preclinical evaluation of new chemical entities as defined by the International Conference of Harmonization Expert Working Group for all drugs in development [15].

## 3. Roadmap for Identifying Regulatory Criteria on Cardiotoxicity Based on Animal Studies

The identification process for setting specific criteria for cardiotoxic chemicals within a regulatory framework should use both animal and human data elaborating the set of findings mentioned above. Human data always have precedence over animal data but are produced or collected after manifestation of the cardiotoxic effects in humans, usually years post-exposure. On the other hand, animal data could be produced in advance and if the appropriate criteria are set, the effects in humans can be accurately predicted based on toxic manifestations in animals.

In order to develop effective criteria, “standard” cardiotoxic substances should be used to produce reference values in animals. Anthracyclines, as explained previously, could represent such a “standard” chemical. Regarding dosage, several schemes have been applied in the literature for the development of cardiotoxicity by anthracyclines in animals, including various dose regimes and different anthracyclines, as well as different animal species [16]. Dosing schemes represent both acute and chronic toxicity and are equally relevant for classification purposes. To monitor cardiotoxicity caused by anthracyclines, cardiac imaging is primarily used and secondarily, biochemical markers. It is important to stress that, for recognizing cardiotoxicity induced by anthracyclines, the specific doses, although recorded, were not essential, since the final effect on heart functioning has been of interest at this stage. At a later stage, dosing could be of importance in identifying the threshold of the adverse effects observed.

In this context, in a previous in-depth review and following a systematic literature search [17], the identification of most used measurements of myocardial function in rats of anthracycline induced cardiotoxicity are presented, together with the range of these values differentiating normal cardiac function from animals with pathological echocardiographic findings indicative of anthracycline cardiotoxicity as per author presentation. At this stage, statistically significant differences are hard to derive, since a meta-analysis has not yet been performed. Therefore, a more descriptive statistical approach of recognizing extreme values and overlapping ranges between exposed and control animals has been applied.

The first attempt to identify a standard animal model focused on rats. Nevertheless, more research is needed to study strain differences as well as different species (i.e., rabbits) with more relevance to humans. The abundance of available studies should also be taken into account, since using already existing data is consistent with animals’ welfare principles.

After having established a “standard” cardiotoxic chemical for reference and having described a range of values representing toxicity for various parameters monitored, the applicability of the identified value ranges should be tested in different substances, such as anabolic androgen steroids (AAS), industrial chemicals, for example metals and pesticides that have been implicated in adversely affecting cardiac pathology causing function impairment.

In addition, the possible mechanisms of cardiotoxicity should also be described and associated with the monitored parameters (histopathological, echocardiographic, biochemical) in a causative way. Hence, information on already-described mechanistic findings of alleged cardiotoxic chemicals could be of relevance.

More specifically, the pathophysiological mechanism of AAS cardiotoxicity is justified since androgen receptors are located both in the endothelium of the vascular smooth muscles and in the myocardium. Although the mode of actions have not been entirely defined, anabolic steroid abuse has been causally linked to effects such as hypertension, myocardial ischemia, left ventricular hypertrophy, sudden cardiac death even in consumers younger than 30 years, heart attack or stroke [18].

With regard to pesticides, the literature data suggest that several modes of action can contribute to cardiotoxicity. More specifically, the most distinguished ones are: inhibition of carboxyl ester hydrolases (Organophosphates/Carbamates), altering the function of voltage-gated sodium channels in insect neuronal membranes, thereby disrupting electrical signaling in the nervous system (Pyrethroids), ligand-gated ion channel activity (GABA-gated chlorine channel blockers) (Organochlorines), cellular hypoxia due to the effect on mitochondria, the inhibition of cytochrome C oxidase and the formation of highly reactive hydroxyl radicals (mitochondrial complex IV electron transport inhibitors) (Phosphides), blocking the cytochrome P450-dependent enzyme C-14 alpha-demethylase, which is needed to convert lanosterol to ergosterol (Triazoles), inhibition of primary events in photosynthesis in the (Photosystem I and II inhibition) (Triazines, Dipyridyl) [19].

Besides, cardiotoxicity with different mechanisms of action has been observed in industrial chemicals. More specifically, metals (e.g., platinum) cause direct injury on the myocytes and cause mitochondrial ultrastructural abnormalities and platelet activation and aggregation. Cobalt causes cardiotoxicity via interference with energy production and contractile mechanisms along with nutrition and hypothyroidism. On the other hand, mercury induces cardiotoxicity by glutathione depletion, the production of ROS and interruption in selenium-dependent endogenous enzymatic reactions. Nanoparticles (i.e., titanium, zinc, silver, carbon, silica and iron oxide nano-materials) induce cardiotoxicity via oxidative stress and inflammation, cellular apoptosis and decreased cell proliferation, decreased heart rate and down regulation of genes, such as Myocyte Enhancer Factor 2C and a homeobox-containing transcription factor NKX2.5, functioning in heart formation and development [20].

Another representative example of an industrial chemical and its relation to cardiotoxicity is Ethanol. Cardiotoxicity outcome involves apoptosis, alterations of the excitation–contraction coupling in cardiomyocytes, structural and functional alterations of the mitochondria and sarcoplasmic reticulum, changes in cytosolic calcium flows, changes in calcium sensitivity of myofilaments, alterations of mitochondrial oxidation, deregulation of protein synthesis, decrease of contractile proteins and disproportion between the different types of myofibrils, changes in the regulation of myosin ATPase, up-regulation of the L-type calcium channels, increase of oxidative stress and the induction of ANP (atrial natriuretic peptide) and p21 (cyclin-dependent kinase inhibitor) mRNA expression in ventricular myocardium [21].

## 4. Evaluation of Preliminary Results in Order to Identify Classification Criteria

### 4.1. Echocardiography Indices

**Figure 1:**
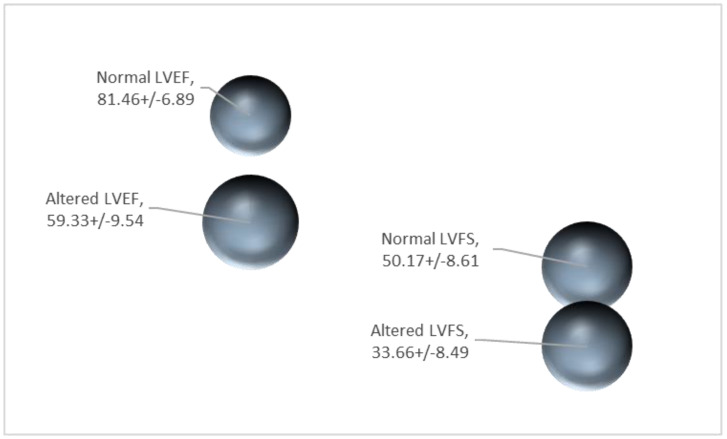
Mean LVEF and LVFS values from control animals (normal) and animals exposed to anthracyclines (altered) (data from [17] not previously published).

Georgiadis et al. (2020) recently published a relevant comprehensive report on echocardiographic data from animal models showing cardiotoxicity with relevance to humans, reviewing anthracyclines [17]. The most common measured indices are left ventricle (LV) fractional shortening (FS) and (LV) ejection fraction (EF) which are indices of left ventricular contractility. In Figure 1 the normal and altered values of the two main echocardiographic indices of anthracycline-treated and control animals, %EF and %FS, respectively, are presented.

There is a clear distinction between the normal LVEF values and the altered ones. More specifically, the normal EF values range from 81.5 ± 6.9% while the altered values range from 59.3 ± 9.5%. It is clear that even in the extreme values, there is no overlap. With regards to the LVFS values, the normal FS values are ranging from 50.17% with a deviation of 8.61% while the altered values are ranging from 33.66% with a deviation of 8.49%. In the case of LVFS there is a small overlap between the normal and altered values (Figure 1).

It should be highlighted that the usual strains used in the rat studies and reviewed in Georgiadis et al. (2020) are equally prone to the cardiotoxic anthracycline potential, regarding the drop of LVEF and LVFS observed.

### 4.2. Biochemical Biomarkers

As a continuation of [17], an in-depth review analysis of several biomarkers altered in the specific animal models after anthracyclines administration, is being performed by our research group (data not yet published) in order to investigate which of them could potentially be used as biochemical criteria in a weight-of-evidence approach together with other lines of evidence, namely the echocardiography indices already presented. The indices for which values are being retrieved from the literature, in the framework of this project, are listed below:

Biomarkers of Oxidative stress

- Catalase (CAT)
- Malondialdehyde (MDA)
- Reactive oxygen species (ROS)
- Superoxide dismutase (SOD)
- Total antioxidant capacity (TAC)
- Total Oxidant Status (TOS)
- Glutathione (GSH)
- Glutathione peroxidase (GSH-Px)
- Lipid hydroperoxide (LH)

Biomarkers relevant to damage of the heart muscle

- Lactate dehydrogenase (LDH)
- Creatine kinase (CK)
- Creatine kinase-myocardial band isoenzyme (CK-MB)
- Cardiac troponin I (cTnI)
- Cardiac troponin T (cTnT)

Biomarkers relevant to increased ventricular blood volume and consequent response of cardiomyocytes to stretching:

- Atrial natriuretic peptide (ANP)
- Brain natriuretic peptide (BNP)

Biomarkers of inflammation

- Interleukin-1 family members (IL-1)
- TNF alpha

**Figure 2:**
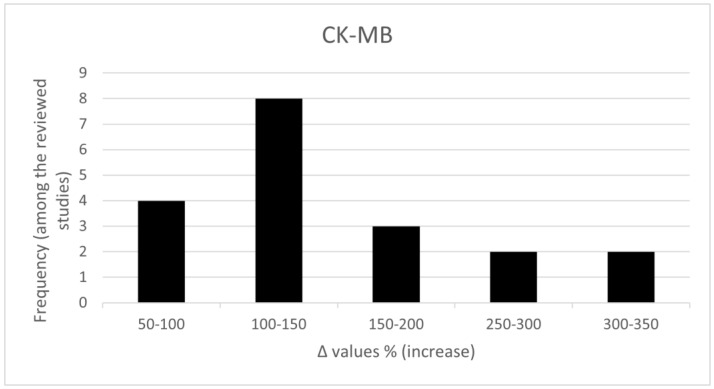
% Increase in CK-MB (%Δ values [(values of rats exposed to anthracyclines) − (values of control rats)] × 100) in rats exposed to anthracyclines compared to control animals, as reported in 19 relevant studies reviewed by Georgiadis et al., 2020 [17] (data from Georgiadis PhD thesis not currently published). %Δ values were calculated in order to overcome the diversity of measuring units used in the literature.

Our preliminary results provided interesting findings. For example, for the important clinically established specific cardiac enzyme, CK-MB, which is used to inform on adverse myocardial events with sufficient sensitivity and specificity in a non-invasive way, the results are shown in Figure 2. The overall increase in CK-MB values of the rats exposed to anthracyclines at well-established cardiotoxic doses, compared to healthy rats, seem to follow the same pattern with respective echocardiographic measures. The vast majority (ca 80%) of the observed CK-MB values in anthracyclines-exposed rats show an increase from 50 to 200% compared to healthy rats (Figure 2). More detailed statistical analysis addressing significant associations is needed at a meta-analysis stage.

**Figure 3:**
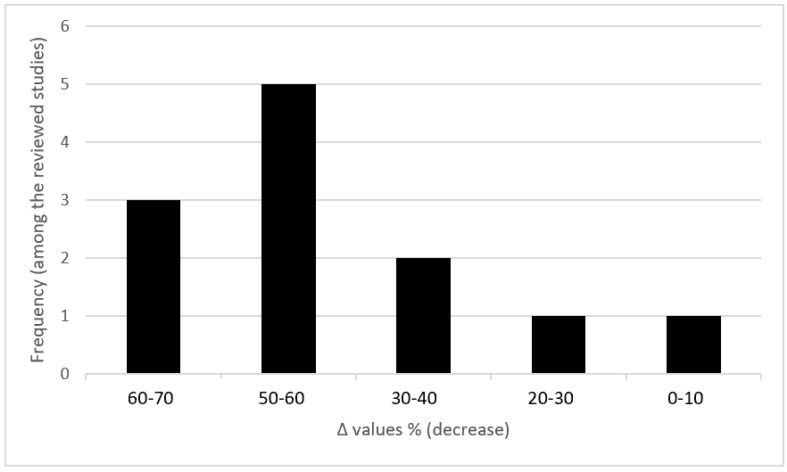
Percentage decrease in cardiac tissue GSH (%Δ values: [(values of rats exposed to anthracyclines) − (values of control rats)] × 100) in rats exposed to anthracyclines compared to control animals as they are reported in 12 relevant studies reviewed by Georgiadis et al. (2020) [17] (data from Georgiadis PhD thesis not currently published). %Δ values were calculated in order to overcome the diversity of measuring units used in the literature.

In addition, generic biomarkers of oxidative stress, both circulating and cardiac-tissue specific, which under certain circumstances could provide supporting evidence in hazard assessment of chemicals for cardiotoxicity, are being reviewed. For example, there is a clear dependence of GSH values in the cardiac tissue upon the anthracycline exposure of rats leading to cardiac dysfunction. More specifically, the overall change in cardiac tissue GSH seems to follow the changes reported for LVEF and shows a decrease ranging from 30 to 70% in the vast majority (83%) of rats with anthracyclines’ caused cardiotoxicity compared to their healthy counterparts (Figure 3). However, it must be noted that whether biochemical markers can be combined with echocardiography and histopathology indices and findings in a scientific assessment is a complex issue and must be taken into account, when the lines of evidence are weighed in the context of a weight of evidence approach.

The statistical analysis of both the cardiac enzymes and of the biomarkers of oxidative stress is on-going. Despite the fact that, at the moment, for some markers the retrieved sample size is small and consequently the statistical power of the analysis is limited, a similar pattern of change is revealed between values of healthy rats and rats with cardiotoxic manifestations due to anthracyclines exposure. This is an important and encouraging finding which, when assessed together with echocardiographic indices and/or histopathological data, can significantly reduce the uncertainty and drastically strengthen the reliability of the weight of evidence assessment for possible cardiotoxicity in humans caused by chemicals.

Therefore, the preliminary results show that more centralized research, preferably coordinated by a regulatory agency, is needed in order to effectively develop the set of classification criteria for cardiotoxicity.

## 5. Future Perspectives and Reflections

The diagnostic methods discussed so far in this manuscript have been frequently used for several years. However, it must be noted that in the past years novel biomarkers of target organ toxicity have been widely used with significant applicability. More specifically, tumor suppressive and oncogenic pathways have been found to involve microRNAs (miRNAs) [22]—microRNAs that are noncoding RNAs that repress the expression of target mRNAs in a post transcriptional way, including apoptosis, differentiation and cancer [23].

The measurement of plasma miRNAs and messenger RNAs (mRNAs) explain the ongoing physiologic processes in cells and tissues that package and release miRNAs into cell-free space. Moreover, miRNAs are non- or minimally-invasive, enhancing animal welfare. Technologically, they are considered ideal for quantitative analysis due to their standardization rapidness and robustness [24].

The aforementioned markers change significantly and early, after their release from tissues into the plasma during toxic events, which shows tissue-specific expression [25]. These advantages have increased the research interest for circulating miRNAs as promising biomarker candidates. They could hopefully play an essential role in human health risk assessment. Another important element is the tissue-specificity and early release of circulating miRNAs upon tissue injury, when damage is still reversible.

Another important novel biomarker is the proto-typic oncogene c-MYC. It is believed that miRNAs linked to c-MYC could be used in human health risk assessment.

Finally, Fatty Acid-Binding Protein (FABP) acts as a long-chain fatty acid carrier in blood and therefore has an essential role in lipid metabolism. The heart type isoenzyme is found in the heart and skeletal muscles and the clinical performance of free FABP is similar to that of myoglobin [26]. It is important to highlight that in a cohort of 19 cancer patients, who underwent immune checkpoint inhibitors therapy (ICIs), FABP levels were increased without significant LVEF reduction, which could indicate that there might be a more sensitive biomarker to detect ICI-related subclinical myocardial damage than traditional cardiac biomarkers [27].

Acknowledging the existence of the aforementioned biomarkers, it must be noted that the current research project of our team aims to facilitate the use of biochemical markers in the hazard assessment of cardiotoxicity. Therefore, it was preferred to limit the review focus in more frequently used and traditional biomarkers that are already found in abundance in the literature and for which it is more potent to find available data. Moreover, the experience of risk assessment for newly appeared hazard classes, such as endocrine disruptors, has shown that, especially for regulatory purposes, the assessors rely on older data for animal welfare reasons. In any case, it must be stressed that, since in the recent years these newly identified biomarkers have been widely implicated in the research of different pathways, it should be further investigated in a different project how they could be applied to the hazard assessment of cardiotoxic chemicals.

In the same line of progress, methods other than two-dimensional echocardiography (2DE) are currently available for LV function quantification, focusing on the early diagnosis of left ventricular dysfunction associated with chemotherapy, including 3-dimensional echocardiogram (3DE), cardiovascular magnetic resonance (CMR), and strain speckle-tracking echocardiogram. However, they require sophisticated technology and advanced medical and technical training compared with 2DE. Historically, multiple-gated acquisition scan (MUGA) has been one of the preferred methods for serial assessment of LVEF while on cancer therapy. LVEF determined by MUGA scan is more accurate and has a greater correlation with other 3-dimensional (3D) imaging modalities (such as CMR) than echocardiography. The greatest limitation of the MUGA scan has been the exposure to radiation, which will cumulate when performing the serial scans during the course of chemotherapy [28]. Regarding monitored parameters of cardiac function, global longitudinal strain (GLS) is a newly emerging topic, which has a significant role in predicting cardiovascular outcomes compared to LVEF. Abnormal GLS is indicative of subclinical left ventricular systolic dysfunction, which for the purposes of regulatory setting of criteria for the early recognition of cardiotoxicity could prove very useful. GLS imaging is underutilized in the detection of subclinical cardiac dysfunction in breast cancer patients receiving chemotherapy [29].

The last but not least criterion, which needs to be investigated and reviewed in the context of a weight-of-evidence approach, includes the findings from histopathological analysis of the heart tissue from animals exposed to well-established cardiotoxic chemicals. Histopathological data, when assessed properly, can provide reliable information. More specifically, significant functional changes in the heart muscle noted at necropsy and/or at microscopic examination, but also morphological, reversible or not, changes which provide evidence of marked heart dysfunction and cell death incapable of regeneration, could be of relevance. Preliminary data in the literature are encouraging. For example, in rabbits exposed to anabolic steroids, local fibrosis and a mild chronic inflammation of cardiac tissue was observed [3,5], while in rabbits exposed to the pesticides propoxur and diazinon the main histopathologic findings were fibrosis, hemorrhagic infiltration of myocardial tissues and degeneration of muscle cells, with no signs of inflammation. What is rather interesting in this case is the persistence or accumulation of different quantities of both pesticides studied in cardiac tissues, showing that the cardiac muscle cells were directly exposed to both pesticides [30]. Finally, clinical observations or small changes in heart weight with no evidence of organ dysfunction could also provide useful information.

In conclusion, more focused research is needed both from scientists and regulators in order to facilitate even further the weight-of-evidence exercise and describe the hazard of cardiotoxicity caused by chemicals with relevance to humans as a regulatorily recognized toxicological class. More specifically, the roadmap suggested in the present manuscript for identifying regulatory criteria from animal studies to include into the regulation consists of the following steps:

1. Identification of the appropriate animal species and strain;
2. Identification of the lines of scientific evidence (e.g., histopathological, biochemical, echocardiographic indices etc.) from animal studies with relevance to humans;
3. Meta-analysis of each line of scientific evidence recognized by animal species after exposure to well-established cardiotoxicants to humans (e.g., anthracyclines) in order to identify threshold values or range of normal and/ or altered values due to exposure;
4. Validation of the above described evidence in animals exposed to other alleged cardiotoxic substances (e.g., AAS and pesticides);
5. Establishment of mechanisms of action based on information either of known or alleged cardiotoxicants and association thereof with the parameters introduced as scientific evidence in the development of classification criteria;
6. Discussion and introduction of novel indices and in silico methods.

## Abbreviations

AAS: anabolic androgen steroids; ALT: Alanine Transaminase; ANP: Atrial natriuretic peptide; AST: Aspartate aminotransferase; BNP: brain natriuretic peptide; CAT: Catalase; CK: creatine kinase; CK-MB: creatine kinase-myocardial band isoenzyme; cTnI: Cardiac troponin I; cTnT: Cardiac troponin T; ECHA: European Chemicals Agency; EFSA: European Food Safety Authority; FABP: Fatty Acid-Binding Protein; FS: fractional shortening; ICIs: Immune checkpoint inhibitors therapy; IL: interleukin; GSH: Glutathione; GSH-Px: glutathione peroxidase; HF: Heart failure; LDH: lactate dehydrogenase; LH: lipid hydroperoxide; LV: left ventricle; MDA: malondialdehyde; miRNAs: microRNAs; ROS: reactive oxygen species; SOD: superoxide dismutase; TAC: Total antioxidant capacity; TNF-α: alpha-Tumor necrosis factor; TOS: Total oxidant status.
